# Quality assessment and chemical diversity of Australian propolis from *Apis mellifera* bees

**DOI:** 10.1038/s41598-022-17955-w

**Published:** 2022-08-09

**Authors:** Chau T. N. Tran, Peter R. Brooks, Tahmikha J. Bryen, Simon Williams, Jessica Berry, Fiona Tavian, Ben McKee, Trong D. Tran

**Affiliations:** 1 School of Science, Technology and Engineering, University of the Sunshine Coast, Maroochydore DC, QLD 4558 Australia; 2 Centre for Bioinnovation, University of the Sunshine Coast, Maroochydore DC, QLD 4558 Australia; 3 Hive and Wellness Australia Pty Ltd, Richlands, QLD 4077 Australia

**Keywords:** Chemistry, Analytical chemistry, Organic chemistry

## Abstract

The propolis industry is well established in European, South American and East Asian countries. Within Australia, this industry is beginning to emerge with a few small-scale producers. To contribute to the development of the Australian propolis industry, the present study aimed to examine the quality and chemical diversity of propolis collected from various regions across Australia. The results of testing 158 samples indicated that Australian propolis had pure resin yielding from 2 to 81% by weight, total phenolic content and total flavonoid content in one gram of dry extract ranging from a few up to 181 mg of gallic acid equivalent and 145 mg of quercetin equivalent, respectively. Some Australian propolis showed more potent antioxidant activity than the well-known Brazilian green, Brazilian red, and Uruguayan and New Zealand poplar-type propolis in an in vitro DPPH assay. In addition, an HPLC–UV analysis resulted in the identification of 16 Australian propolis types which can be considered as high-grade propolis owing to their high total phenolic content. Chemometric analysis of their ^1^H NMR spectra revealed that propolis originating from the eastern and western coasts of Australia could be significantly discriminated based on their chemical composition.

## Introduction

Honey bee propolis is a resinous material from plant exudates mixed with saliva and beeswax by European honey bees (*Apis mellifera*) belonging to the family Apidae^1^. This apiary product has been used since 300 B.C. in the folk medicine of many cultures as a natural antibiotic and wound healing agent^2^. Before the 1990s, research publications about propolis were occasional^3^. However over the last three decades the interest in propolis chemistry and its pharmacological properties has grown^3^. Besides the antimicrobial property, propolis shows a wide spectrum of biological activities including antioxidant, anti-inflammation, antidiabetics, dermatoprotection, antiallergic, immunomodulation and anticancer activities^4^. Propolis is in growing demand with diverse products including propolis-enriched honey, propolis candies, propolis tincture, mouth and throat sprays, soaps, toothpastes and skincare creams^5^.

In general, raw propolis consists of plant resin, beeswax, and some minor constituents, including pollen, minerals and dead bees^6^. Its primary biological activities are derived from the constituents in the resin^3^. So far, over five hundred compounds including 79.5% polyphenolic, 18.9% terpenoids and 1.6% fatty acid-derived compounds have been identified in honey bee propolis^3^. As most honey bee propolis components are naturally found in food, they are already recognized as safe substances for food-related product developments^7^. In addition, propolis is a ready source of drug discovery and development for the treatment of infectious and chronic diseases owing to more than 90% of the compounds exhibiting oral bioavailability property and fitting within the chemical space of drug-like molecules^3^. A randomized and controlled clinical trial study for 124 hospitalized adult patients with COVID-19, revealed that adjunct treatment with standardized Brazilian green propolis extract significantly reduced the hospital stay post-intervention^8^.

Plant source significantly affects the chemical profile of propolis and defines the different types found^9^. In temperate regions, poplar bud exudates (*Populus* spp.) have been shown to be the common source of propolis. Poplar propolis is the most studied and the best known type of propolis, both from a chemical and pharmacological point of view^9^. Poplar propolis contains high levels of flavonoids (chrysin, pinocembrin and galangin) and caffeic acid esters (caffeic acid phenethyl ester and its derivetives)^3^. In tropical areas, Brazilian green and red propolis are two well-studied and renown propolis types^10^. Green propolis is derived from the apical buds and young leaves of the Brazilian native plant *Baccharis dracunculifolia*, and comprises mainly prenylated phenylpropanoids such as artepillin C (3,5-diprenyl-*p*-coumaric acid), and chlorogenic acid derivatives^10^. Red propolis is collected from a red exudate of *Dalbergia ecastaphyllum* stems and is a source of the isoflavonoids, biochanin A and medicarpin^10^. Additionally, honey bees are reported to also source resin from other plant genera *Acacia, Anacardium, Araucaria, Azadirachta, Betula, Bursera, Cistus, Clusia, Lepidosperma, Liquidambar, Macaranga, Pinus, Styrax, Xanthorrhoea* and *Zuccagnia*^3^.

**Figure 1:**
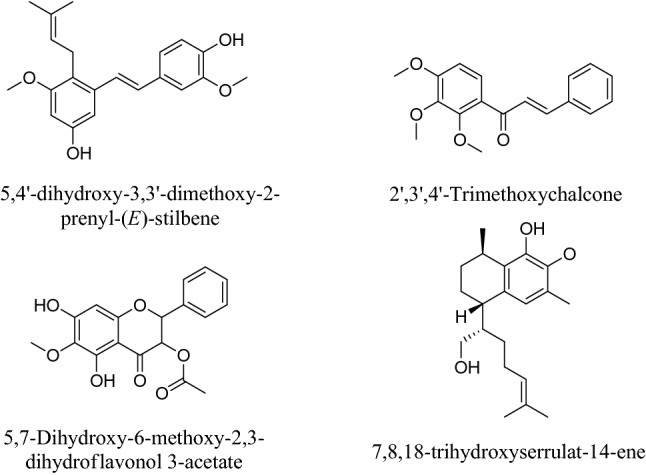
Some new compounds isolated from Australian propolis (adapted from Abu-Mellal et al.^13^, Tran et al.^12^, and Aminimoghadamfarouj et al.^15^).

As a result of geographic isolation and a vast array of geographical and environmental habitats, Australia is classified as one of the most megadiverse countries in the world with 84% of the endemic terrestrial plant species accounting for 6% of global plant species^11^. This native flora is a unique and diverse source for Australian propolis. Therefore, Australia has the capability to produce unique and premium propolis types. However, studies on Australian propolis are limited to the investigation of samples from South Australia, which identified some new and known flavonoids^12^, prenylated cinnamates^13^, stilbenes^13,14^, and diterpenes^15^ (Fig. 1). Owing to novel stilbenes coming from the endemic plant species *Lepidosperma* sp.^14^, propolis from Kangaroo Island in South Australia is considered as a unique propolis type in the world^3^. The Kangaroo Island propolis extract exhibited four times more potent antioxidant activity than the Brazilian green propolis^13^. A stilbene compound, 5,4′-dihydroxy-3,3′-dimethoxy-2-prenyl-(*E*)-stilbene (Fig. 1), found in the Kangaroo Island propolis inhibited the growth of a panel of cancer cell lines more potently than the anticancer drug tamoxifen^14^.

Currently, only small-scale propolis production occurs in Australia, mainly in South Australia. There remains a lack of understanding of the chemical composition of Australian propolis and its therapeutic potentials. Considered a nuisance hive product by beekeepers, Australian propolis is regularly discarded^16^. With approximately 530,000 honey bee hives^17^, it is estimated that Australia can secure millions of dollars with the domestic harvesting of propolis. The domestic harvesting will provide extra income for both Australian beekeepers and processors while reducing the reliance on imported propolis^16^. To provide a brief overview of the quality of Australian propolis, this study assessed the resin recovered yield, total phenolic content (TPC), total flavonoid content (TFC), and antioxidant activity of the collected propolis. The assessment utilised the key criteria used to develop the quality standards for propolis in Brazil, Russia, Portugal, Japan, Korea, China, and Taiwan^18–20^. The chemical diversity of Australian propolis was also examined from the analysis of their HPLC–UV and ^1^H NMR profiling data.

## Results

**Figure 2:**
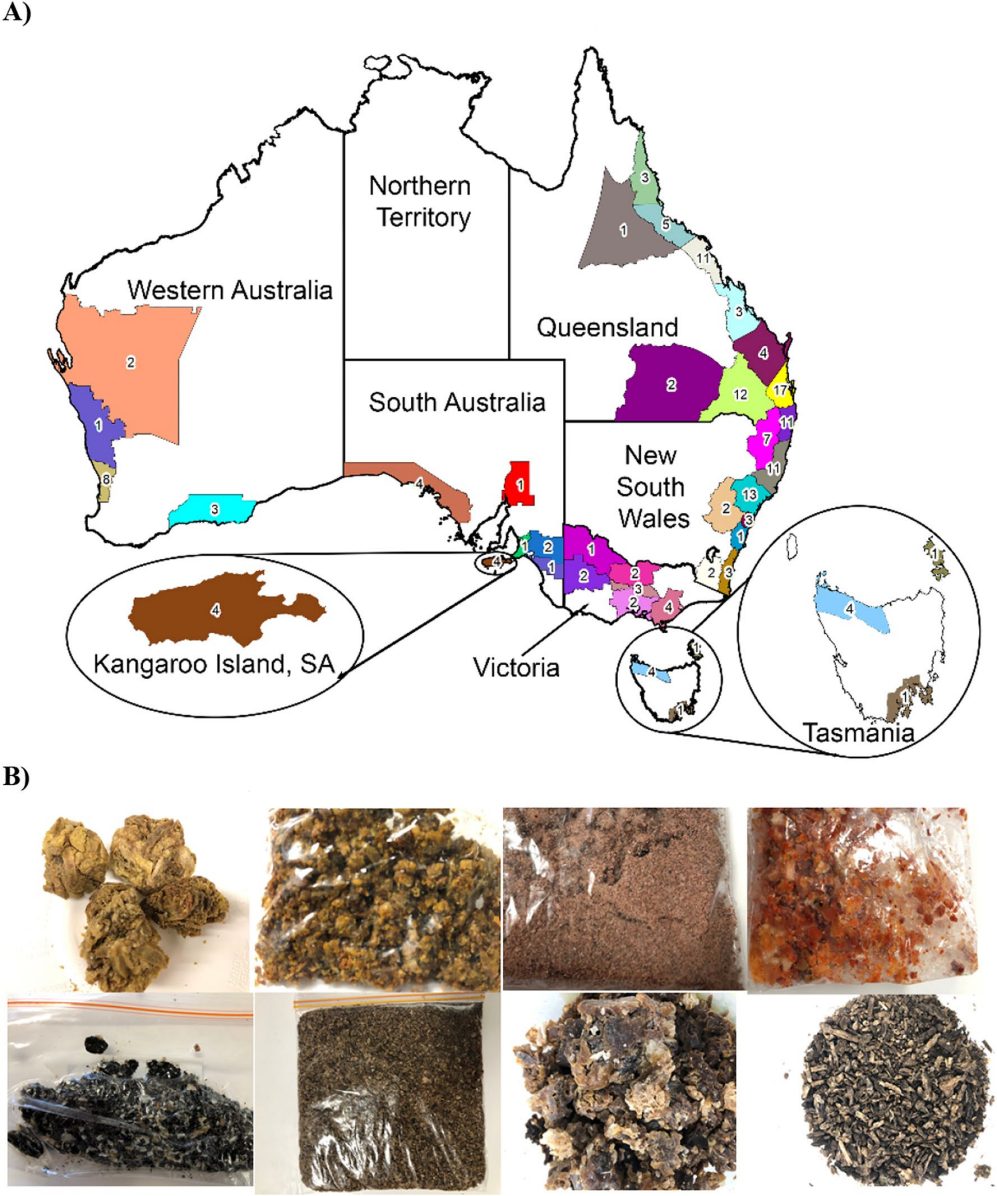
(**A**) Map of Australia showing propolis collection sites; (**B**) Photos of Australian honey bee propolis with diverse colour.

In this study, 158 raw propolis samples were collected from different regions across six Australian states by local beekeepers from 2018 to 2020 (Fig. 2A). All Australian samples showed variations in appearance with colours including brown, dark brown, black, red, red-brown, orange or yellow, and aroma ranging from strongly resin to sweet honey (Fig. 2B). In addition, Brazilian green and red propolis, as well as Uruguayan and New Zealand poplar-type propolis were used as referenced samples for the quality and chemical diversity assessments.

### Assessment of resin recovered yield

In general, the aim of propolis extraction is to dissolve bioactive constituents in resin and eliminate beeswax^21^. Literature indicates most antioxidant propolis compositions solubilise better in ethanol and methanol than other solvents such as water, ethyl acetate, chloroform, and benzene^21^. However, the organic solvents can also dissolve beeswax with the amount dependent on solvent polarity^18^. Therefore, it was suggested to use co-solvents of ethanol–water or methanol–water for propolis extraction^22^. Ethanol is safer to handle over methanol, making it the preferred solvent for propolis extraction in research and industrial settings. The optimal concentration of ethanol in water was found to be between 70–95% ethanol, with 70–80% ethanol often used to extract propolis compounds with minimal wax contamination^21^. This study used 70% ethanol in water to extract raw propolis.

**Figure 3:**
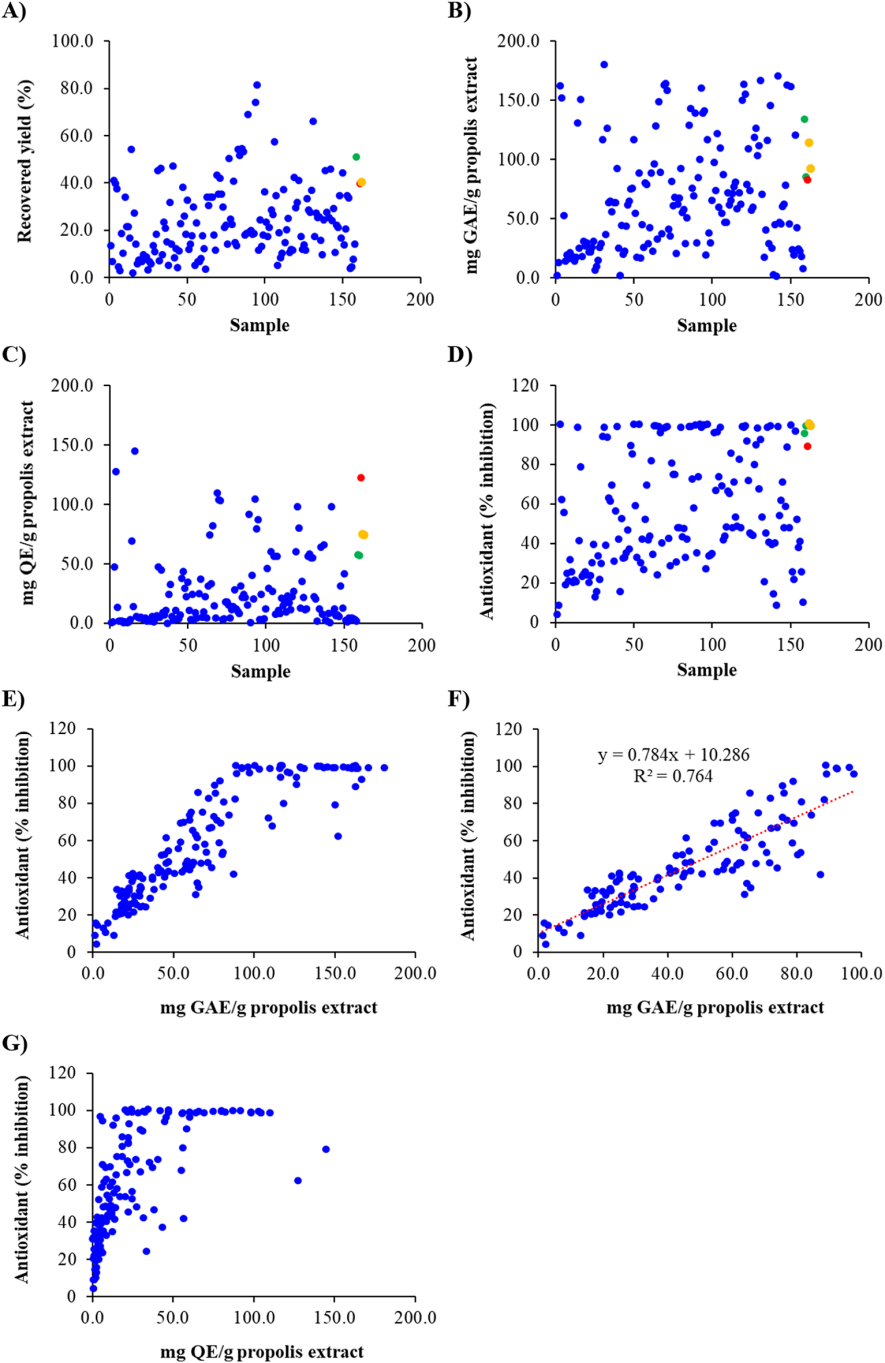
Recovered yield (**A**), total phenolic content (**B**), total flavonoid content (**C**) and antioxidant activity (**D**) of Australian propolis and other referenced propolis; correlations between antioxidant activity and total phenolic content of Australian propolis (**E**,**F**); and a correlation between antioxidant activity and total flavonoid content of Australian propolis (**G**) (Blue: Australian propolis; Green: Brazilian green propolis; Red: Brazilian red propolis; and Orange: Uruguayan poplar propolis; New Zealand poplar propolis was excluded in these analyses since the sample was commercialised as a liquid extract whose solvent could not be evaporated).

The resin recovered yield of 158 samples was highly variable, ranging from 2 to 81% (Fig. 3A). About 6% of the Australian propolis samples exceeded the resin yield of the Brazilian green propolis (51%). At the same time, 15% exceeded the Brazilian red (40%) and Uruguayan poplar (40%) propolis yield. There were 66 samples with a yield above the average of 23%. The remaining 92 samples had lower levels of pure resin due to higher levels of wax and other compounds insoluble in ethanol 70%. In addition, drought and bushfire conditions occurring in Australia during 2019 and 2020 might also have a negative effect on the resin recovered yield. The data also revealed old propolis samples often exhibited lower yields compared to fresh samples. Here old propolis samples were defined as being stored at room temperature for a period greater than a year by beekeepers before sending for analysis.

### Assessment of total phenolic content

Polyphenols or phenolic compounds are the most abundant secondary metabolites in plants^23^. In honey bee propolis, nearly 30 classes of phenolic compounds, have been identified^3^. They consist of both flavonoids (mainly flavanone and flavone) and non-flavonoids (mainly phenylpropanoid)^3^. Chemically, phenolic compounds are good electron donors that can disrupt the oxidation of organic radicals by reacting with the oxygen and nitrogen species present on the radical^24^. Due to their antioxidant activity, phenolics are beneficial against inflammation, diabetes, cardiovascular, and neurodegenerative diseases, mutagenesis and carcinogenesis^24,25^.

TPC of Australian propolis extracts in this study ranged from 1.3 to 180.5 mg GAE/g extract with a mean value of 68 mg GAE/g extract (Fig. 3B). Australian propolis contained TPC relatively comparable to Brazilian, Croatian, Moroccan and Palestinian propolis and much lower TPC than studies where poplar propolis was the dominant type in the country (Table S1, Supplementary information). However, it is not easy to compare the TPC values in this study with others due to different assay protocols and reagent ratios used in different labs. By including the Brazilian green, Brazilian red, and Uruguayan poplar propolis as references, an overview of the phenolic level in Australian propolis can be assessed undoubtedly (Fig. 3B). The referenced propolis had TPC ranging from 82.7 to 134.4 mg GAE/g extract (Table 1), and 13% of the Australian samples had TPC over the upper limits of this range.

**Table 1:** The top 10% of Australian propolis (n = 16) ordered in increasing antioxidant activity compared to Brazilian green, Brazilian red and Uruguayan poplar propolis.

Sample	Recovered yield (%)	Total phenolic content (mg GAE/g extract)	Total flavonoid content (mg QE/g extract)	Antioxidant activity IC_50_ (µg/mL)
1	41 ± 1	162.6 ± 4.9	47.3 ± 0.3	7.3 ± 0.2
2	42 ± 2	158.4 ± 2.1	103.0 ± 1.0	10.0 ± 0.4
3	66 ± 1	167.0 ± 1.1	22.6 ± 0.7	10.0 ± 0.2
4	45 ± 5	180.5 ± 0.5	47.2 ± 1.4	10.2 ± 0.4
5	30 ± 0	163.4 ± 0.7	98.0 ± 3.3	10.5 ± 0.1
6	43 ± 2	163.2 ± 1.6	109.8 ± 0.7	10.6 ± 1.0
7	74 ± 2	139.5 ± 0.3	79.7 ± 1.5	10.6 ± 0.1
8	43 ± 3	150.3 ± 0.4	60.3 ± 1.9	10.6 ± 0.3
9	19 ± 4	160.7 ± 1.5	104.5 ± 1.1	10.7 ± 0.3
10	35 ± 5	164.5 ± 2.0	104.2 ± 2.4	10.9 ± 0.5
11	81 ± 4	141.2 ± 1.2	87.1 ± 1.8	12.1 ± 0.2
12	44 ± 2	161.5 ± 1.1	41.8 ± 1.1	12.3 ± 0.2
13	32 ± 2	155.1 ± 0.5	80.1 ± 3.0	12.4 ± 0.1
14	46 ± 0	170.7 ± 0.2	98.4 ± 0.9	14.2 ± 0.1
15	54 ± 2	131.1 ± 2.4	69.2 ± 0.4	14.4 ± 0.2
16	69 ± 1	139.5 ± 1.9	91.9 ± 3.2	14.4 ± 0.2
Brazilian green_1	51 ± 1	134.4 ± 2.0	57.6 ± 1.1	23.5 ± 2.7
Brazilian green_2	N.D	85.5 ± 0.7	56.8 ± 2.2	21.2 ± 0.7
Brazilian red_3	40 ± 3	82.7 ± 0.5	122.3 ± 3.8	6.8 ± 0.2
Uruguay_1	40 ± 3	114.5 ± 1.8	75.0 ± 0.8	7.7 ± 0.3
Uruguay_2	N.D	92.5 ± 0.7	74.3 ± 2.1	18.6 ± 0.5

*N.D* not determined as samples were commercialised as a liquid extract.

### Assessment of total flavonoid content

Among over 8000 phenolic compounds found from plants, half of them are flavonoids presenting as aglycone, glycosides and methylated derivatives^23^. Most flavonoids identified from propolis are aglycone and methylated analogues, while only three flavonoid glycosides have been isolated so far^3^. China suggests the TFC as a reference standard for their propolis which is mainly the poplar propolis type^26^. While Chinese legislation requires a minimum flavonoid content of 8% (w/w) in raw propolis^27^, Brazilian legislation accepts 0.5% (w/w) as a minimum^6^. Studies indicated that the poplar propolis typically contained higher total flavonoid content than the Brazilian green propolis^28^.

Owing to a specific chemical scaffold of a carbonyl C-4 in the B-ring and a hydroxy group at C-5 in the A-ring, flavonoids can bind strongly to aluminum ions to form yellow stable complexes [Al^III^(flavonoid–H)_2_]^+^ showing an electronic band at a wavelength of 415 nm^29^. The flavanone-aluminum complex has been known to display less absorbance than the flavone-aluminum complex^30^. Often, the measurement of the yellow [Al^III^(flavonoid–H)_2_]^+^ complex provides quantification of TFC in natural sources, expressed in an equivalent term of milligrams of quercetin^20,31–43^.

The assessment of Australian propolis indicated the TFC of Australian propolis was in the range of 0.2–144.8 mg QE/g extract with a mean value of 24.0 mg QE/g extract. Argentina^37^, Croatia^42^, Japan^39^, Morocco^19^, Palestine^19^, and South Korea^40^ also have TFC reported within the range recorded for Australian propolis (Table S1, Supplementary information). Within the control samples, Brazilian red propolis had the highest TFC with 122.3 mg QE/g extract while the Uruguayan poplar and Brazilian green propolis contained 75 and 57 mg QE/g extract on average, respectively (Fig. 3C). There were 2, 13 and 20 Australian propolis samples having higher TFC than the Brazilian red, Uruguayan poplar and Brazilian green propolis, respectively (Fig. 3C). From the assessment of the total phenolic and flavonoid contents, it can be seen that Australian propolis samples have the wide variation of both phenolic and flavonoid levels which reflect the relatively diverse range of Australian plant resins the honey bees forage.

### Assessment of free radical scavenging activity

The formation of reactive oxygen and nitrogen species has been known to associate with the oxidative deterioration of food products and human diseases caused by oxidative stress processes such as atherosclerosis, diabetes mellitus, chronic inflammation, neurodegenerative disorders, and cancer^44–46^. In the present work, the scavenging capacity and reducing power of 2,2-diphenyl-1-picrylhydrazyl (DPPH) free radical was chosen for the antioxidant evaluation of propolis. The DPPH radical scavenging activity of the Australian propolis extracts varied between 4 to 100% inhibition at 100 µg/mL (Fig. 3D). Samples with TPC over 100 mg GAE/g extract showed 80–100% inhibition at 100 µg/mL (Fig. 3E). A positive linear correlation was observed between the DPPH radical scavenging property and TPC when TPC values were below 100 mg GAE/g extract (Fig. 3F). The results of this study agree with the data from a previous study reported by Kasote et al.^47^, confirming that propolis with high levels of phenolic compounds shows a strong antioxidant activity. However, there was no clear positive correlation between TFC and the DPPH radical scavenging activity (Fig. 3G).

To compare the antioxidant potency, IC_50_ values of those propolis samples showing 100% inhibition at 100 µg/mL were determined. The results indicated that the antioxidant activity of some Australian propolis was comparable or better than the Brazilian green, Brazilian red, and Uruguayan poplar propolis (Table 1). Notably, these Australian propolis samples displayed significantly higher TPC. While they all showed less TFC than the Brazilian red propolis, over 50% of them had more TFC than the Brazilian green and Uruguayan poplar propolis (Table 1).

### HPLC profiling and classification of high-grade propolis

For pharmaceutical and food applications, knowledge of the chemical composition of raw materials is necessary^48^. Chromatography and spectroscopy are often utilised for the qualification, traceability and authentication of raw materials^49^. Numerous studies on the chemical composition of propolis by various techniques including thin layer chromatography (TLC)^50^, high performance TLC^51,52^, high-performance liquid chromatography (HPLC)^53–55^, liquid chromatography coupled with mass spectrometry (LC–MS)^56–58^, gas chromatography coupled with mass spectrometry^59^, near infrared spectroscopy^60^, and nuclear magnetic resonance (NMR)^26,43,47,49,61–65^ have been reported. Among them, HPLC has proved to be an effective and reliable technique in the separation of complex natural product mixtures and routine analysis^48,55,66^. Based on retention times and the UV adsorption spectra of the peaks in a chromatogram, HPLC–UV is often selected as a profiling method to provide a quick classification of propolis samples without detailed identification of individual components^53–55^. This study classified propolis samples into two groups. High-grade propolis (57 samples) had TPC greater or equal to 75 mg GAE/g extract (10% over the TPC mean value). Low-grade propolis (101 samples) consisted of the remaining samples. Defining Australian high-grade propolis allows the prioritisation of samples for future developments.

**Figure 4:**
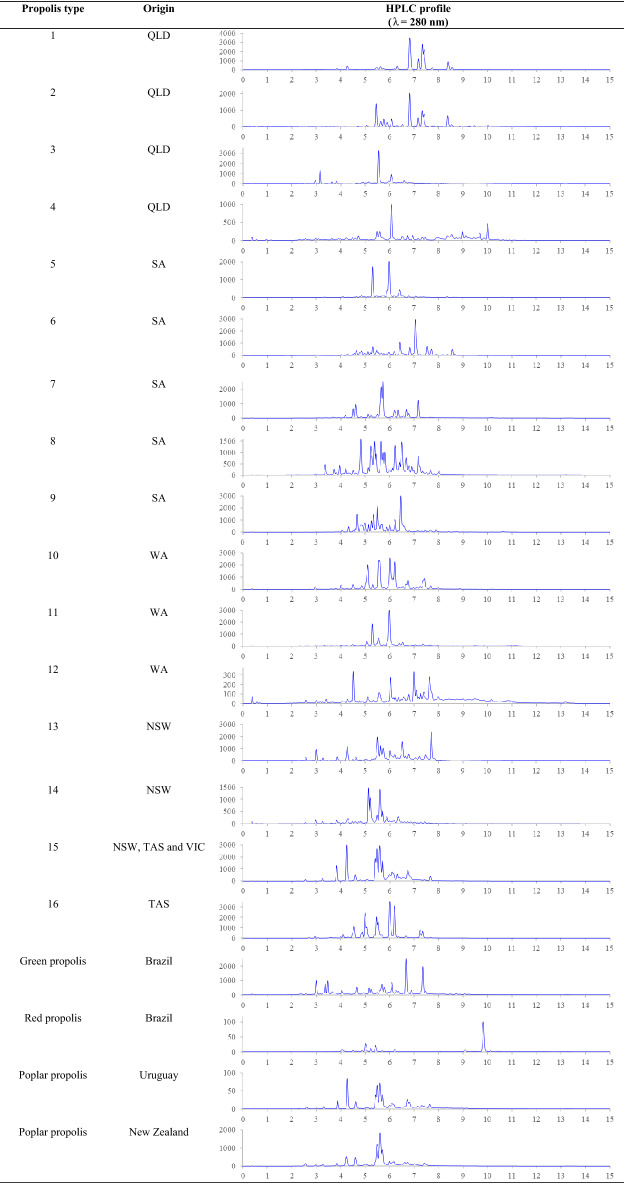
HPLC profiles of 16 Australian high-grade propolis types and propolis references.

Most flavonoids and phenolics show two absorption maximums at around 240–285 and 300–400 nm, which correspond to the benzoyl and the cinnamoyl systems, respectively^67,68^. Due to the high molar absorptivity of the different phenolic classes, 280 nm is the most generic wavelength for phenolic analysis using HPLC–UV^18,48,68,69^. By profiling the chemical composition of 57 high-grade propolis extracts, 16 high-grade propolis types were identified (Fig. 4). These Australian propolis types showed TPC, TFC and antioxidant activity relatively competitive to the three referenced propolis (Table S2, Supplementary information). The HPLC profiles revealed that propolis types 4–12 and 16 were unique and characteristic for each state while types 1–3 and 13–15 shared some similarities. In particular, types 1–2 contained similar compounds eluting from 6.8 to 8.6 min. Compared to type 1, type 2 displayed more polar compounds in a region of 5.1–6.1 min while type 3 had other extra compounds between 2.9 and 3.8 min. A similar case occurred for propolis types 13–15, which shared five compounds eluting at 4.2, 4.6, 5.5, 5.6 and 5.7 min. The results indicated these propolis types were likely produced from multiple resin sources. Surprisingly, the chromatographic profile of the Australian high-grade propolis type 15 was almost identical to that of Uruguayan and New Zealand poplar propolis (Fig. 4). This finding provides evidence that Australia also produces poplar propolis similar to other countries in its temperate regions (NSW, TAS and VIC). Whether *Populus* spp. or other plant species is the botanical source of this Australian propolis type warrants further investigation. The Brazilian green and red propolis are considered premium propolis types, and they represent two of 12 propolis types defined in Brazil^70^. No Australian propolis tested in this study showed similar HPLC profiles to that of Brazilian green and red propolis. However, the identification of 16 Australian phenolic-rich propolis types indicates that Australia is also home to a diverse range of world-class propolis.

### ^1^H NMR profiling and chemometric analysis of Australian propolis

**Figure 5:**
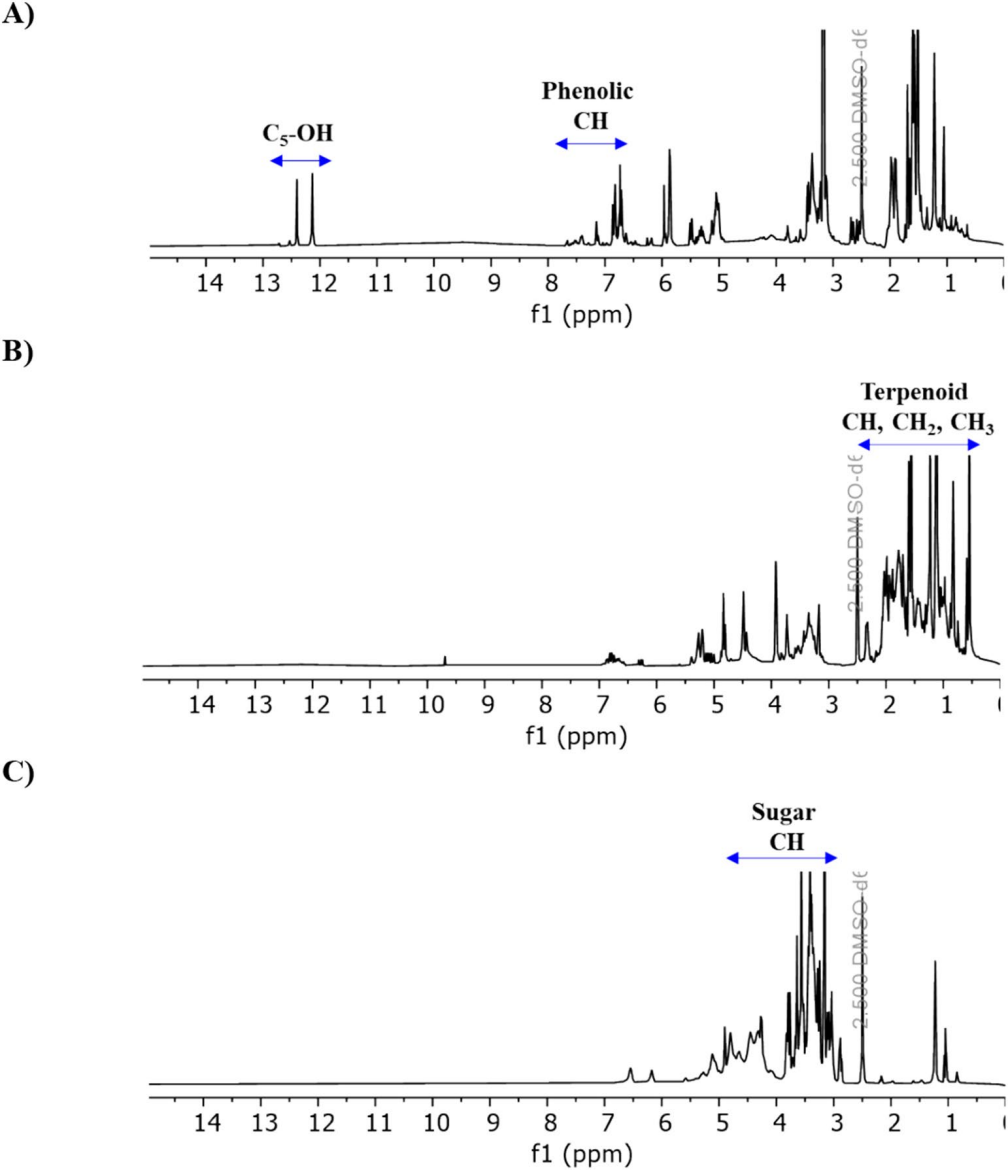
^1^H NMR spectra of some Australian propolis in the study: (**A**) phenolic-rich propolis, (**B**) terpenoid-rich propolis, and (**C**) sugar-rich propolis.

Recently ^1^H NMR has been receiving considerable attention for metabolic fingerprinting analysis to test food quality, origin, manufacture, or authenticity due to its high reproducibility, unbiased structural information production and possibility of detecting fraudulent compounds in a sample^71^. This non-destructive technique has been utilised to evaluate and identify possible adulterations in coffee^72,73^, honey^74,75^, milk and dairy products^76–78^, liquor^79–82^, oil^83,84^ and juice^85,86^. For propolis, the advantage of the ^1^H NMR method is that it can detect waxes and terpenoids, which are generally not observed by HPLC–UV due to their lack of UV chromophores^62^. In general, the ^1^H NMR spectra of propolis extracts display all phytochemical compound classes, which can be determined based on their NMR fingerprints present in specific regions of the spectra. The peaks that occur in the region 0.5–3.0 ppm are mainly terpenoids, steroids or linear fatty acids from wax residues, whereas peaks found in regions 3.5–5.5 and 6.5–8.0 ppm are sugars and phenolics, respectively^47^. The singlet around *δ*_H_ 11.0–12.0 ppm could be attributed to an intramolecular hydrogen bond formed by the –OH group at C-5 and the ketone at C-4 of the flavonoid molecules^43^. However, this signal is only observable in the ^1^H NMR spectrum when the sample is recorded in aprotic deuterium solvents^87^. Nine studies utilised the ^1^H NMR profiling method to examine the chemical diversity of propolis associated with seasonal variations or different regions in Brazil, China, India, Mexico and several European countries (Table 2). Three NMR solvents, including DMSO-*d*_*6*_, MeOH-*d*_*4*_ and CHCl_3_-*d* were used in these studies. Among them, DMSO-*d*_*6*_ was the most selected solvent due to its advantages in solubilising a wide range of compounds with different polarities and facilitating the detection of exchangeable proton signals, which is beneficial for the assignment of flavonoids^88^. Therefore, DMSO-*d*_*6*_ was also chosen in this study to relatively support the level of TPC and TFC determined. With the profiling of 158 samples, this study has generated the largest ^1^H NMR propolis database so far (Table 2). The ^1^H NMR analysis confirmed that all high-grade propolis contained characteristic phenolic signals, particularly from 6.50–7.80 ppm, with relatively higher intensities than other non-phenolic signals. In addition, the typical proton signal at 11.0–12.0 ppm was mostly observed in the ^1^H spectra of propolis having high total flavonoid content. Propolis in the low-grade group were mainly terpenoid-rich or sugar-rich as assigned by high terpenoid or sugar fingerprint signals (Fig. 5).

**Table 2:** ^1^H NMR based metabolomic analysis of propolis in different studies.

Sample origin	Number of samples	NMR solvent	Study
Brazil	59	MeOH-*d*_*4*_	Maraschin et al.^61^
China	63	CHCl_3_-*d*	Wang et al.^26^
Europe, Asia, Africa, Brazil, and Solomon Islands	43	CHCl_3_-*d*/MeOH-*d*_*4*_ (2:1)	Watson et al.^62^
Greece	20	MeOH-*d*_*4*_	Stavropoulou et al.^63^
India	19	MeOH-*d*_*4*_	Kasote et al.^47^
Italy	65	DMSO-*d*_*6*_	Bertelli et al.^64^
Italy	60	DMSO-*d*_*6*_	Papotti et al.^65^
Mexico	39	DMSO-*d*_*6*_	Rivero-Cruz et al.^43^
Serbia, Bosnia and Herzegovina, and Bulgaria	59	DMSO-*d*_*6*_	Andelkovic et al.^49^
Australia	158	DMSO-*d*_*6*_	This study

**Figure 6:**
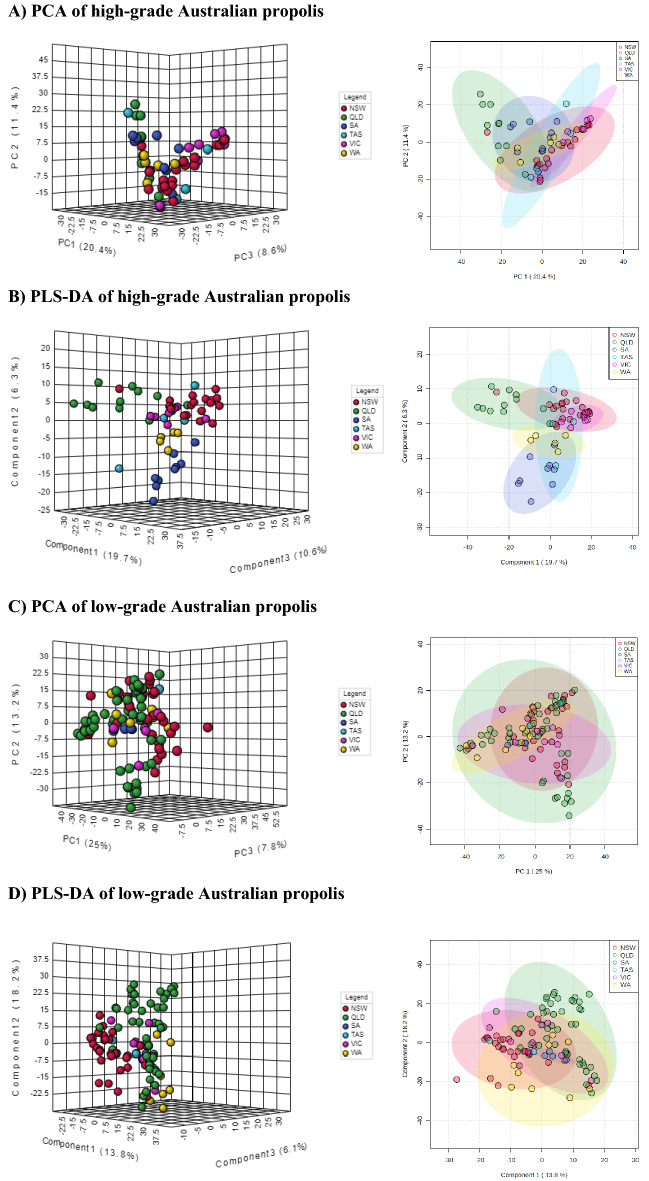
The 3D and 2D score plots of PCA and PLS-DA analyses for high-grade and low-grade Australian propolis with Hotelling’s T2 ellipses present a 95% confidence level (Red: New South Wales; Green: Queensland; Blue: South Australia; Cyan: Tasmania; Purple: Victoria; and Yellow: Western Australia).

The ^1^H NMR spectral data of both high-grade and low-grade propolis samples were further processed and analysed by chemometric methodologies using untargeted multivariate statistical principal component analysis (PCA) and partial least squares—discriminant analysis (PLS-DA) to understand chemical similarity or differences according to the geographical regions the samples were collected. The PCA explains in an unsupervised way the variance of each dataset when increasing the number of principal components without referring to any class label while the PLS-DA extracts the information that can predict all possible class memberships from linear combinations of original NMR bins with the use of multivariate regression techniques and assess all class discriminations^71^. The PCA score plots of high-grade propolis did not show any separate grouping among six datasets with a 95% confidence level (Fig. 6A). In contrast to the PCA, the supervised PLS-DA (Fig. 6B) provided some discriminations enabling the differentiation of QLD, SA and WA from other clusters. In particular, the separated Hotelling’s T2 ellipses of QLD and SA clusters indicated the uniqueness in chemical compositions of their high-grade propolis (Fig. 6B). Since the SA and WA states are adjacent (Fig. 2A), they shared some similar propolis types but also had some distinct propolis as shown in Fig. 6B. The results also revealed that chemical constituents of propolis from NSW and VIC were relatively similar. Interestingly, despite having a small number of samples in the dataset, the TAS cluster spread across other states, indicating this state possesses highly diverse propolis samples, some of which are similar to NSW and VIC propolis. The conclusion for the chemical diversity of TAS propolis becomes more certain if additional samples are discovered. The PCA of the low-grade propolis did not show any significant discriminations (Fig. 6C). Further PLS-DA analysis only exhibited a partial separation of some propolis in QLD and WA (Fig. 6D). This discrimination reflected the greater geographic and floral differences between QLD and WA, located on Australia's eastern and western coasts, respectively (Fig. 2A).

In conclusion, by utilising chemical assays with HPLC and ^1^H NMR profiling, this study introduced an overview of the Australian propolis’s quality and chemical diversity. Although the production cost of Australian propolis might be greater compared to international sourced propolis, targeting premium propolis market is a strategy for Australia to establish a propolis industry. The data from this study indicates it is possible to find premium Australian propolis types which have higher total phenolic content, total flavonoid content and antioxidant property than some well-known international propolis. The study has identified and reported 16 types of Australian high-grade propolis for the first time. These findings will contribute to the premiumisation of Australian honey bee propolis. In addition, the ^1^H NMR database generated in this study will help address the issues of adulterated, unnaturally enhanced and faux propolis in the future for the Australian propolis industry. Although more work is necessary to build a comprehensive picture for Australian propolis regarding their chemical composition and therapeutic values, the results of this study provide a foundation for the commercial production of the new premium propolis in the domestic and global food, and cosmeceutical industries.

## Materials and methods

### Solvents and reagents

All solvents including ethanol (EtOH), methanol (MeOH) and acetonitrile (MeCN) used for extraction and chromatography were HPLC grade and purchased from Merck. The water (H_2_O) used was Milli-Q water. Folin–Ciocalteu 2 N solution, sodium carbonate (Na_2_CO_3_), gallic acid (≥ 95%), aluminium nitrate (Al(NO_3_)_3_), potassium acetate (CH_3_COOK), quercetin (≥ 95%), 2,2-diphenyl-1-picrylhydrazyl (DPPH), dimethylsulfoxide-*d*_*6*_ (DMSO-*d*_*6*_) and formic acid were purchased from Sigma-Aldrich.

### Sample collection and extraction

The study used propolis samples produced by the European honey bee (*Apis mellifera*). Raw Brazilian green propolis and lyophilized Brazilian green propolis extract were purchased from Apiario Diamante, Brazil. Raw Brazilian red propolis was purchased from MN Própolis. Raw Uruguayan propolis and propolis liquid extract were purchased from Apiter SA, Montevideo, Uruguay. New Zealand propolis liquid extract was purchased from Comvita, New Zealand. Australian raw propolis samples were collected from across Australia by beekeepers including New South Wales (NSW, 53 samples), Queensland (QLD, 58 samples), South Australia (SA, 13 samples), Tasmania (TAS, 6 samples), Victoria (VIC, 14 samples) and Western Australia (WA, 14 samples) and stored in darkness at 4 °C. Frozen raw propolis sample was powdered by grinding. Fine propolis powder (0.5 g × 3) was mixed with 5 mL of 70% (v/v) ethanol solution, heated at 65 °C for 30 min and then extracted in an ultrasonic bath for 5 min. The sample was placed in ice for 10 min before being centrifuged at 3600 rpm at 4 °C for 10 min. The supernatant was dried down under vacuum using a GeneVac EZ-2 evaporator to obtain dry propolis extract. Each extract was prepared in MeOH at a concentration of 10 mg/mL for total phenolic content, total flavonoid content and antioxidant assays, and HPLC analysis. For NMR analysis, the extract was prepared in DMSO-*d*_*6*_ at a concentration of 50 mg/mL.

### Determination of total phenolic content

The total phenolic content of propolis extracts was determined by the Folin–Ciocalteu method^18^. All phenolics are oxidized by the yellow Folin-Ciocalteu reagent to form a blue complex under basic conditions and can be quantified by visible-light spectrophotometry at a wavelength of 760 nm^89^. In brief, 80 µL of the Folin–Ciocalteu solution was added to 410 µL of H_2_O, followed by the addition of 10 µL of propolis extracts (10 mg/mL) or 10 μL of gallic acid standard solution in MeOH and then 500 µL of 10% (m/v) aqueous sodium carbonate. MeOH was used as a blank sample (negative control). The samples in Eppendorf tubes were incubated in darkness at room temperature for 60 min before being plated to a 96-well plate (200 µL/well). Finally, the absorbance at 760 nm was measured on a Perkin Elmer Enspire microplate reader. All measurements were performed in triplicates, the mean values were interpolated in a gallic acid calibration curve and the total phenolic content was expressed as mg Gallic Acid Equivalents (GAE) per gram of dry extract^19,31–43,89^.

### Determination of total flavonoid content

The total flavonoid content of propolis extracts was determined by the aluminium colorimetric assay as previously described with some modifications^30^. Briefly, 20 µL of 10% Al(NO_3_)_3_ in H_2_O and 20 µL of 1.0 M CH_3_COOK in H_2_O were added to 950 µL of MeOH and then mixed with 10 µL of propolis extracts (10 mg/mL) or standard solution of quercetin in MeOH. MeOH was used as a blank sample (negative control). The samples were incubated in darkness at room temperature for 45 min before being plated to a 96-well plate (200 µL/well). The absorbance was measured at 415 nm on a Perkin Elmer Enspire microplate reader. All measurements were performed in triplicates, the mean values were interpolated in a quercetin calibration curve and total flavonoid content was expressed as mg Quercetin Equivalents (QE) per gram of dry extract.

### Evaluation of antioxidant activity using DPPH free radical scavenging assay

The DPPH free radical scavenging activity of the propolis extracts at different concentrations was evaluated using the DPPH assay as described previously with some modifications^31^. Briefly, the DPPH solution was prepared on the day of measuring at a concentration of 100 µM in MeOH. The propolis extracts (200 μL) at different concentrations were added to 600 μL of DPPH solution in Eppendorf tubes. The mixtures were kept in the dark at room temperature for 20 min before being plated to a 96-well plate (200 µL/well) and measured at 518 nm using a Perkin Elmer Enspire microplate reader. All evaluations were performed in triplicates. Gallic acid and MeOH were used as positive and negative controls. The % inhibition of the DPPH radical for each sample was normalised and calculated using the following formula:

$$\%\, Inhibition = \left[ {1 - \frac{{\left( {A_{S} - A_{P} } \right)}}{{\left( {A_{B} - A_{P} } \right)}}} \right] \times 100$$

where A_S_ is the absorbance of the sample, A_P_ is the absorbance of the positive control and A_B_ is the absorbance of the blank sample (negative control).

The IC_50_ values of the extracts were determined as the concentration required to inhibit 50% of DPPH free radical.

### HPLC analysis

The HPLC analysis was performed on the HPLC system (Agilent 1290) equipped with auto-sampler, quaternary pump, and DAD detector. Each sample (10 mg/mL in MeOH) was injected (2 µL) onto a C_18_ uHPLC Zorbax column (2.1 × 50 mm, 1.8 µm) and eluted by H_2_O (0.1% formic acid) and MeCN (0.1% formic acid) as mobile phases A and B, respectively. Detection was achieved at 280 nm. The mobile phases were used at a flow rate of 0.4 mL/min with a 15-min gradient program which was started at 2% B for 0.5 min, increased to 100% B for 9.0 min, kept at this level for the next 3.0 min, then reduced to 2% B for 1 min and finally re-equilibrated for 1.5 min.

### NMR analysis and processing

The ^1^H NMR spectra were acquired at 300 K on a Bruker Ascend 400 MHz spectrometer equipped with a 5 mm room temperature probe and processed by Bruker TopSpin 3.6 software. The spectrum was recorded using the standard pulse sequence, with a 90° pulse length of 9.61 µs, 64 scans, a spectral width of 16 ppm, a relaxation delay of 5 s, and an acquisition time of 3.75 s. The spectra were referenced to the DMSO residual solvent signal *δ*_H_ 2.50 ppm.

### Chemometric analysis

The ^1^H NMR spectral data between 0.00–8.00 ppm in an Excel format were exported to MetaboAnalyst 5.0 software^90^ (https://www.metaboanalyst.ca/) for principal component analysis (PCA) and partial least-squares discriminant analysis (PLS-DA). In all cases, Hotelling’s T2 regions depicted by ellipses in score plots of each model defined a 95% confidence interval.

## Supplementary Information

Supplementary Information.

## Data Availability

All data generated or analysed during this study are included in this published article and its Supplementary Information file.
