# Supplementary material for: Quality assessment and chemical diversity of Australian propolis from Apis mellifera bees
Source: Sci Rep. 2022 Aug 9;12:13574. doi: 10.1038/s41598-022-17955-w (PMC9362168; doi:10.1038/s41598-022-17955-w)
Supplement: Supplementary file 1 — Supplementary Information. [file 41598_2022_17955_MOESM1_ESM.docx]

**Quality assessment and chemical diversity of Australian propolis from *Apis mellifera* bees**

Chau T. N. Tran,^1^ Peter R. Brooks,^1,2^ Tahmikha J. Bryen,^1^ Simon Williams,^1^ Jessica Berry,^3^ Fiona Tavian,^3^ Ben McKee,^3^ and Trong D. Tran^1,2,^*

^1^ School of Science, Technology and Engineering, University of the Sunshine Coast, Maroochydore DC, Queensland 4558, Australia.

^2^ Centre for Bioinnovation, University of the Sunshine Coast, Maroochydore DC, Queensland 4558, Australia.

^3^ Hive and Wellness Australia Pty Ltd, Richlands, Queensland 4077, Australia.

* Tel: +61 7 5459 4579. Email: ttran1@usc.edu.au.

**Supporting information**

**Table S1.** Total phenolic content and total flavonoid content of propolis in different studies.

| **Country** | **Number of samples** | **Total phenolic content**  **(mg GAE/g extract)** | **Total flavonoid content**  **(mg QE/g extract)** | **Study** |
| --- | --- | --- | --- | --- |
| Algeria | 6 | 55 – 279 | 10 – 69 | Boufadi et al.^1^ |
| Argentina | 10 | 257 – 393 | 66 –133 | Lima et al.^2^ |
| Argentina | 17 | 14 – 348 | 0 – 256 | Kumazawa et al.^3^ |
| Argentina | 30 | 42 – 253 | 2 – 28 | Chaillou et al.^4^ |
| Brazil | 12 | 94 – 149 | 6 – 21 | Schmidt et al.^5^ |
| China | 20 | 43 – 302 | 8 – 188 | Ahn et al.^6^ |
| Croatia | 7 | 14 – 190 | 7 – 104 | Svecnjak et al.^7^ |
| Japan | 14 | 53 – 431 | 19 – 113 | Hamasaka et al.^8^ |
| Morocco | 3 | 115 – 148 | 77 – 118 | Touzani et al.^9^ |
| Mexico | 35 | 12 – 287 | 1 – 57 | Rivero-Cruz et al.^10^ |
| Palestine | 2 | 75 – 136 | 27 – 107 | Touzani et al.^9^ |
| South Korea | 6 | 85 – 283 | 16 – 136 | Ahn et al.^11^ |
| South Korea | 20 | 49 – 239 | 21 – 50 | Wang et al.^12^ |
| Australia | 158 | 1 – 181 | 0 – 145 | This study |

**Table S2.** Total phenolic content, total flavonoid content and antioxidant activity of 16 Australian high-grade propolis types

| **Propolis type** | **Total phenolic content**  **(mg GAE/g extract)** | **Total flavonoid content**  **(mg QE/g extract)** | **Antioxidant activity**  **IC_50_ (µg/mL)** |
| --- | --- | --- | --- |
| 1 | 75.0 – 180.5 | 12.6 – 47.3 | 7.3 – 52.0 |
| 2 | 89.2 – 117.1 | 14.5 – 24.1 | 26.6 – 31.6 |
| 3 | 116.5 – 120.4 | 4.7 – 6.2 | 21.5 – 28.5 |
| 4 | 81.4 | 18.2 | 53.2 |
| 5 | 92.4 | 28.0 | 40.3 |
| 6 | 129.2 – 143.1 | 21.6 – 24.6 | 21.1 – 22.3 |
| 7 | 111.5 – 126.5 | 55.1 – 58.3 | 50.6 – 97.5 |
| 8 | 167.0 | 22.6 | 10.0 |
| 9 | 163.1 | 31.3 | 53.8 |
| 10 | 75.5 – 109.1 | 21.9 – 35.3 | 61.2 – 81.5 |
| 11 | 96.3 | 31.4 | 22.4 |
| 12 | 78.6 | 6.1 | 71.7 |
| 13 | 76.8 – 131.1 | 17.2 – 69.2 | 14.4 – 94.2 |
| 14 | 80.3 | 24.8 | 100 |
| 15 | 75.4 – 170.7 | 29.6 – 144.8 | 10.0 – 68.3 |
| 16 | 126.5 | 44.7 | 20.6 |
| Brazilian green propolis | 85.5 – 134.4 | 56.8 – 57.6 | 21.2 – 23.5 |
| Brazilian red propolis | 82.7 | 122.3 | 6.8 |
| Uruguayan poplar propolis | 92.5 – 114.5 | 74.3 – 75.0 | 7.7 – 18.6 |

**References**

1 Boufadi, Y. M. *et al.* Characterization and antioxidant properties of six Algerian propolis extracts: ethyl acetate extracts inhibit myeloperoxidase activity. *Int. J. Mol. Sci.* **15**, 2327-2345, doi:10.3390/ijms15022327 (2014).

2 Lima, B. *et al.* Main flavonoids, DPPH activity, and metal content allow determination of the geographical origin of propolis from the province of San Juan (Argentina). *J. Agric. Food Chem.* **57**, 2691-2698, doi:10.1021/jf803866t (2009).

3 Kumazawa, S., Ahn, M.-R., Fujimoto, T. & Kato, M. Radical-scavenging activity and phenolic constituents of propolis from different regions of Argentina. *Nat. Prod. Res.* **24**, 804-812, doi:10.1080/14786410802615270 (2010).

4 Chaillou, L. L. & Nazareno, M. A. Bioactivity of propolis from Santiago del Estero, Argentina, related to their chemical composition. *LWT - Food Sci. Technol.* **42**, 1422-1427, doi:https://doi.org/10.1016/j.lwt.2009.03.002 (2009).

5 Schmidt, E. M. *et al.* A comparison between characterization and biological properties of Brazilian fresh and aged propolis. *BioMed Res. Int.* **2014**, 257617, doi:10.1155/2014/257617 (2014).

6 Ahn, M.-R. *et al.* Antioxidant activity and constituents of propolis collected in various areas of China. *Food Chem.* **101**, 1383-1392, doi:https://doi.org/10.1016/j.foodchem.2006.03.045 (2007).

7 Svečnjak, L., Marijanović, Z., Okińczyc, P., Marek Kuś, P. & Jerković, I. Mediterranean propolis from the Adriatic Sea Islands as a source of natural antioxidants: Comprehensive chemical biodiversity determined by GC-MS, FTIR-ATR, UHPLC-DAD-QqTOF-MS, DPPH and FRAP assay. *Antioxidants* **9**, 337, doi:10.3390/antiox9040337 (2020).

8 Hamasaka, T., Kumazawa, S., Fujimoto, T. & Nakayama, T. Antioxidant activity and constituents of propolis collected in various areas of Japan. *Food Sci. Technol. Res.* **10**, 86-92, doi:10.3136/fstr.10.86 (2004).

9 Touzani, S. *et al.* Determination of phenolic compounds in various propolis samples collected from an African and an Asian region and their impact on antioxidant and antibacterial activities. *Molecules* **26**, 4589, doi:10.3390/molecules26154589 (2021).

10 Rivero-Cruz, J. F. *et al.* Prediction of antimicrobial and antioxidant activities of Mexican propolis by ^1^H-NMR spectroscopy and chemometrics data analysis. *Molecules* **22**, 1184 (2017).

11 Ahn, M.-R., Kumazawa, S., Hamasaka, T., Bang, K.-S. & Nakayama, T. Antioxidant activity and constituents of propolis collected in various areas of Korea. *J. Agric. Food Chem.* **52**, 7286-7292, doi:10.1021/jf048726s (2004).

12 Wang, X. *et al.* Relationship between total phenolic contents and biological properties of propolis from 20 different regions in South Korea. *BMC Complement. Altern. Med.* **16**, 65, doi:10.1186/s12906-016-1043-y (2016).
